# Infection-free rates and Sequelae predict factors in bone transportation for infected tibia: a systematic review and meta-analysis

**DOI:** 10.1186/s12891-018-2363-5

**Published:** 2018-12-13

**Authors:** Zhen Zhang, W. Benton Swanson, Yan-Hong Wang, Wei Lin, Guanglin Wang

**Affiliations:** 1 Department of Orthopedics, West China Hospital, Sichuan University, No. 37, Guoxue Lane, Wuhou District, Chengdu, 610041 Sichuan Province China; 2 Department of Biologic and Materials Sciences, School of Dentistry, University of Michigan, Ann Arbor, USA; 3 Department of Neonatology, Beijing Gynecology & Obstetrics Hospital, Capital Medical University, Beijing, China; 4 Department of Gynecology, West China Second Hospital, Sichuan University, Chengdu, China

**Keywords:** Bone transportation, Infection-free rate, Predict factor of Sequelae

## Abstract

**Background:**

Tibia infected nonunion and chronic osteomyelitis are challenging clinical presentations. Bone transportation with external or hybrid fixators (combined external and internal fixators) is versatile to solve these problems. However, the infection-free rates of these fixator systems are unknown. Additionally, the prognosis factors for results of bone transportation are obscure. Therefore, this systematic review and meta-analysis was conducted to answer these questions.

**Methods:**

A systematic review was conducted following the PRISMA-IPD guidelines. Relevant publications from January 1995 to September 2018 were compiled from Medline, Embase, and Cochrane. The infection-free rates of external and hybrid fixators were achieved by synthesizing aggregate data and individual participant data (IPD). IPD was analyzed by two-stage method with logistical regression to identify prognosis factors of sequelae.

**Results:**

Twenty-two studies with 518 patients were identified, including 11 studies with 167 patients’ IPD, and 11 studies with 351 patients’ aggregate data. The infection-free rate of hybrid fixator group was 86% (95%CI: 79–94%), lower than that of external fixator which was 97% (95%CI: 95–98%,). The number of previous surgeries was found predict factor of bone union sequelae (*p* = 0.04) and function sequelae(*p* < 0.01); The external fixation time was found predict factor of function sequelae (*p* = 0.015).

**Conclusions:**

Hybrid fixators may be associated with a greater risk of infection-recurrence in the treatment of tibia infected nonunion and chronic osteomyelitis. The number of previous surgeries and external fixation time can be used as predictors of outcomes. Proper fixators and meticulously designed surgery are important to avoid unexpected operations and shorten external fixation time.

## Background

Tibial infected nonunion and chronic posttraumatic osteomyelitis are common clinical presentations which pose substantial burdens on both patients and society [1, 2]. However, their treatment remains a large challenge; most cases are associated with infection caused by antibiotic-resistant bacteria, bone and soft tissue loss, deformities, and limb-length discrepancy [3, 4]. Many patients suffer from multiple operations due to more than one stage of treatment and associated complications [5, 6], especially the reoccurring infection which may be refractory and lead to amputation [7–9]. To achieve an infection-free result, radical debridement is necessary, but massive skeletal defects also result as a consequence [9]. Bone transport, based on principles of distraction osteogenesis, could tackle segmental bone defects and coexisting problems of lone bone infection simultaneously. The procedure of bone transportation could be divided into distraction and consolidation phases: After corticectomy in metaphysis, the lost tissue is compensated by gradual distraction of healthy bone segment towards the defect site, and consequent consolidation follows when bone ends meet [10, 11].During the phases of distraction and consolidation in bone transportation technique, osseous stability is provided by various fixator systems [12].

However, factors related to fixator choice for infected tibia is still obscure [8, 13]. Among fixators, the most commonly used are external frames including circular and mono-lateral fixators. The external frames allow for early weight bearing and maintenance of tibia length during treatment. Nevertheless, external fixators suffer from complications associated with long-time external fixation, such as pin site infection and joint stiffness [14]. To shorten the external fixation time, several researchers have combined internal fixators with external frames for bone transportation during distraction and/or consolidation phases [13, 15–19]. This “hybrid fixator” system facilitates early removal of the external frame, helps maintain alignment, and prevents refracture [17, 20]. Despite its advantages, the hybrid fixators are suspected to be associated with a greater risk of infection recurrence which is worrisome for both clinicians and patients [8, 21]. During treatment procedure, infection recurrence leads to repetitive debridement, prolonged treatment time, and increased psychological stress on patients. Patients suffered from multiple reinfection may even refuse further revision, demanding amputation as the final solution [22]. Even though the infection-free result is important in this scenario, the infection-free rates of external and hybrid fixators are still unknown.

Additionally, Ilizarov methods are associated with high rates of temporary complication and residual sequela which are difficult to avoid. As an application of distraction osteogenesis, bone transport technique were also reported with sequelae in many studies [6, 23]. Residual sequelae, which remain unsolved at the end of the treatment period, are used as indicators for criteria to grade outcomes of both bone union and function [3, 24, 25]. Despite the high rate of satisfactory results (excellent and good) reported in most studies, the rate of sequela-free result (excellent), is varied. Since prognosis factors are seldom studied, it is difficult to determine those factors leading to a sequela-free result.

Thus, this systematic review was conducted to addresses the question in the treatment of tibial infected nonunion and chronic osteomyelitis.: 1) Do hybrid fixators have lower incidence of infection-free results compared to external fixators? 2) What are predictive factors of sequelae in bone transportation technique?

## Methods

### Strategy

The systematic review was conducted according to Preferred Reported Items for Systematic Reviews of Meta-Analyses Statement for Individual Patient Data (PRISMA-IPD) [26]. Databases including Medline, Embase, and Cochrane were searched from January 1995 until September 2018. Key words “bone transport technique,” “Ilizarov,” “infectious non-union,” “osteomyelitis,” “distraction osteogenesis,” and “tibia” were combined in the search procedure. The reference lists of included studies were manually searched to avoid omissions.

### Eligibility criteria

After excluding duplicates, two independent reviewers screened all remaining records based on both titles and abstracts, then screened the full text of the potentially relevant studies. Studies were considered acceptable for inclusion if the following criteria were fulfilled: (1) studies treated adult patients (more than 16 years of age) diagnosed of tibia infectious non-union or osteomyelitis; (2) studies with a minimum sample of 5 aforementioned consecutive patients were treated with multifocal bone transport technique; For IPD collection, each subgroup of fixator systems should contain no less than 5 patients. (3) main outcome of bone union and function were graded to excellent, good, fair or poor according to Paley or ASAMI classification, and recurrence of osteomyelitis or deep bone infection was recorded; (4) original articles written in English. In the cases of research on the same patient group published at the same institution, the most complete or recent data was used. Disagreements were solved by consulting a third reviewer.

### Data collection

Specific information from selected papers was compiled. According to the details of Paley or ASAMI classification, the outcomes are graded by the number of sequelae: “excellent” is the outcome without sequelae, while the “good,” “fair,” and “poor” outcomes are associated with increasing numbers of sequelae. Herein we define those patients who received excellent results in a “sequelae free” group, while the good, fair, and poor are considered “sequelae.” IPD were compiled for the following variables where available: demographic information (age, gender), number of previous operations, type of fixator, size of bone defect, length of distraction osteogenesis, time of external fixation and consolidation, healing index (time between application of fixators and consolidation divided by the length of the defect), and external fixation index (external fixation time divided by the length of defect).

### Statistical analysis

The rates of infection-free outcomes were synthesized in subgroups of external fixator group or hybrid fixator group with the variance-stabilizing double arcsine transformation [27]. Heterogeneity was quantified using the *I*^2^ statistic. The *I*^2^ heterogeneity was degreed as follows: < 25% low, 25 to 50% moderate and > 50% high. Fixed-effects models were used for low and moderate heterogeneity while random-effects models for high heterogeneity. To understand the factors which impact associated sequelae, two-step method with logistic regression was used to investigate the IPD (95% confidence interval). IPD from each study was independently analyzed in the first step to produce an estimate for each study, and then these data were analyzed. All analyses were performed using Stata (version 14.0, StataCorp, College Station, TX, USA).

## Results

**Fig. 1:**
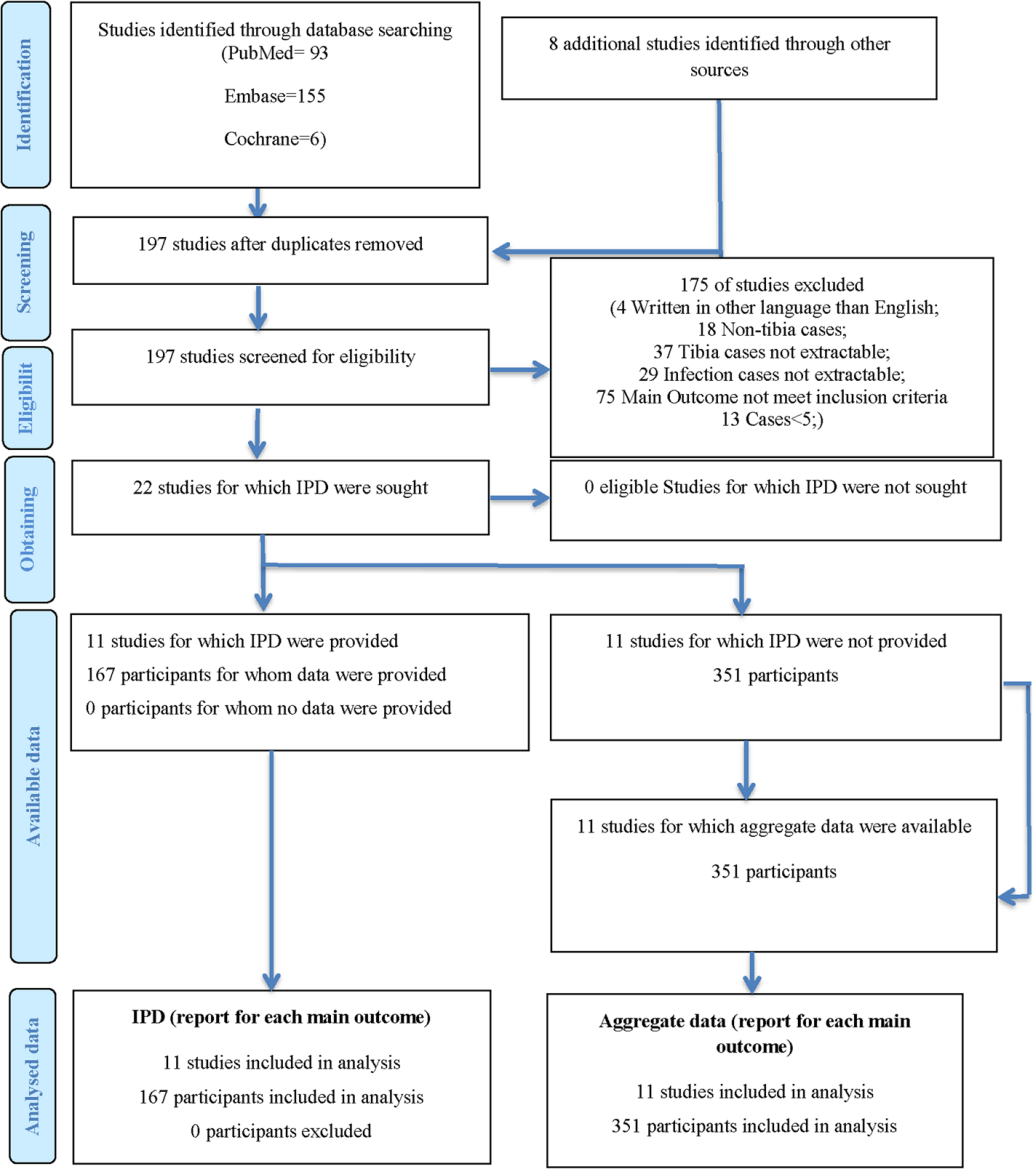
PRISMA-IPD flow diagram, illustrating the identification, screening and exclusion process

**Fig. 2:**
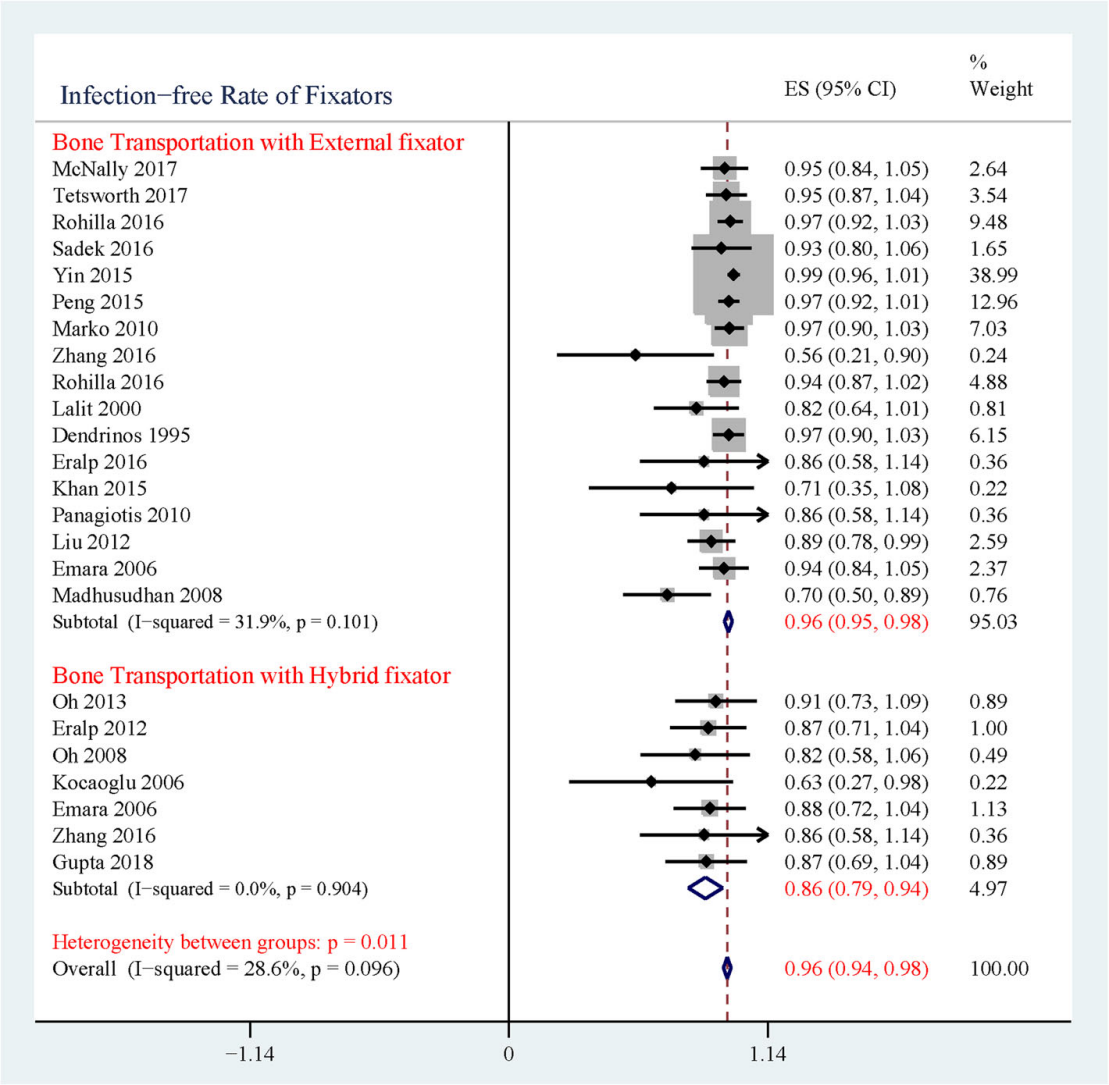
Forest plot of infection-free rate of fixators used in bone transportation

Based on our review, twenty-two studies with 518 patients met inclusion criteria: eleven studies with 167 patients’ IPD, and 11 studies with 351 patients’ aggregate data (Fig. 1). Hybrid fixators were applied on 63 patients, while external fixators were applied on 454 patients (Table 1). The overall infection-free rate of bone transportation for tibia infected nonunion and chronic osteomyelitis is 96% (95%CI: 94–98%, Fig. 2). Significant heterogeneity existed between the groups of hybrid and external fixators (*p* = 0.01, Fig. 2). The infection-free rate of hybrid fixator group was 86% (95%CI: 79–94%, Fig. 2), while that of external fixator was 97% (95%CI: 95–98%, Fig. 2).

**Table 1:** Summary of Included Studies

ID	Data type	Patients Number	Gender		Age(year)	Fixator System	Soft-tissue Reconstruction	Satisfactory Result		Sequelae-free		Infection Free
			Male	Female				Bone	Function	Bone	Function	
Dendrinos 1995 [38]	IPD	28	23	5	37.43(18~74)	Ilizarov EF	–	24	18	14	7	28
Eralp 2016 [43]	IPD	6	3	3	48.33(33~79)	Ilizarov EF; TSF	1^b^	–	4	–	2	6
Khan 2015 [44]	IPD	6	6	0	40.60(19~55)	Ilizarov EF	3^b^	4	4	0	1	5
Kocaoglu 2006 [17]	IPD	7	5	2	35.29(18~52)	Hybrid Fixator	2^b; c^	7	7	6	6	5
Lalit 2000 [40]	IPD	16	16	0	30.81(17~46)	Ilizarov EF	–	11	12	8	5	14
Liu 2012 [45]	IPD	35	25	10	37.29(18~64)	EF	5^b^	33	34	28	30	32
Marko 2010 [46]	IPD	30	29	1	30.57(20~49)	Ilizarov EF	–	29	27	19	13	30
Oh 2008 [16]	IPD	10	10	0	46.00(18~76)	Hybrid Fixator	5^b^	–	10	–	4	9
Oh 2013 [47]	IPD	10	9	1	40.40(16~64)	Hybrid Fixator	4^b^	10	9	10	6	10
Panagiotis 2010 [48]	IPD	6	5	1	34.50(21~52)	Ilizarov EF	2^b^	6	4	3	1	6
Zhang 2016 [19]	IPD	14	13	1	38.07(21~62)	Mono-lateral EF; Hybrid Fixators^a^	1^b^;2^c^	14	11	12	8	5;6^a^
Emara 2008 [18]	AD	33	22	11	29	Ilizarov ring; Hybrid Fixators^a^	–	33	28	32	25	16;16^a^
McNally 2017 [22]	AD	18	–	–	–	Ilizarov ring	–	14	17	13	13	18
Peng 2015 [49]	AD	58	38	20	29.4(18~51)	Ilizarov ring	–	53	46	30	28	57
Rohilla 2016 [4]	AD	70	62	8	31.25(18~65)	Mono-lateral EF; Ring EF	0	62	55	35	28	35
Sadek 2016 [5]	AD	14	12	2	29.50	Ring EF	8	14	8	11	8	14
Tetsworth 2017 [7]	AD	21	18	3	38.2(18~66)	Ring/Ilizarov EF	–	20	20	15	14	21
Tong 2017 [50]	AD	13	–	–	–	Mono-lateral EF; Ilizarov EF	–	10	5	5	1	9
Yin 2014 [51]	AD	72	–	–	–	Ilizarov EF	–	63	52	46	25	72
Eralp 2012 [13]	AD	15	14	3	39(25~69)	Hybrid Fixator	–	14	15	10	10	14
Gupta 2018 [52]	AD	14	13	1	38.1	Hybrid Fixator	1^b^	14	14	14	8	13
Madhusudhan 2008 [53]	AD	22	–	–	–	Ilizarov EF	4^b^	13	5	5	1	16

*AD* Aggregate data, *IPD* Individual participant data, *EF* External Fixators, *TSF* Taylor Spatial Frame; a: Early change from external fixators to internal fixators; b: Soft-tissue flap; c: Skin graft

For the IPD data, the mean age of patients was 38.25 years (range from 16 to 79 years), the mean number of previous surgeries was 3.49 (range from 1 to 20). The mean size of bone defect after debridement was 5.25 cm (range from 1 to 12 cm). The mean healing index was 1.74 months/cm (range from 0.8 to 15.8 months/cm), the mean external fixator index was 1.1 months/cm (range from 0.4 to 1.7 months/cm) (Table 2).

**Table 2:** Summary of IPD

ID	Previous Surgery	Bone defect(cm)	Bone graft	Length of distraction(cm)	External Fixation Time (weeks)	External Fixation Index (months/cm)	Heal Time (weeks)	Heal Index (months/cm)
Dendrinos 1995	4.04(1~20)	6.14 ± 2.68	3	5.54 ± 2.46	41.46(21.50~77.40)	1.95 ± 0.74	27.77(17.20~43.00)	1.35 ± 0.64
Eralp 2016		–	–	3.00 ± 2.61	32.26(21.43~42.86)	1.04 ± 0.11	–	–
Khan 2015	2.00(1~3)	2.82 ± 0.95	–	2.82 ± 0.95	–	–	72.80(32.00~124.00)	7.77 ± 6.38
Kocaoglu 2006	5.29(1~20)	7.00 ± 1.83	7	–	12.63(8.00~20.00)	–	33.09(21.45~55.77)	1.07 ± 0.21
Lalit 2000	2.38(1~5)	7.71 ± 2.30	3	–	–	–	–	–
Liu 2012	2.69(1~6)	3.55 ± 1.47	2	7.81 ± 2.21	–	–	–	1.37 ± 0.12
Marko 2010	–	6.87 ± 1.76	1	6.53 ± 1.53	–	1.48 ± 0.07	17.86(14.00~20.00)	1.03 ± 0.06
Oh 2008	–	5.75 ± 2.89	10	–	–	0.80 ± 0.27	–	2.09 ± 0.61
Oh 2013	–	5.91 ± 1.96	10	–	–	0.45 ± 0.08	–	2.15 ± 0.23
Panagiotis 2010	5.00(1~6)	6.67 ± 3.20	–	–	33.67(17.00~59.00)	–	–	–
Zhang 2016	4.36(1~7)	–	6	5.91 ± 1.24	41.43(23.00~57.00)	–	44.86(31.00~61.00)	1.87 ± 0.59

Among the variables of IPD, the number of previous operations before bone transportation had a significant impact on the sequelae of both bone union (*p* = 0.04) and function (*p* < 0.01). Longer external fixation (*p* = 0.015) was associated with a greater chance of functional sequelae. Age, size of bone defect, and length of bone distraction did not have significant impacts on the sequelae of bone union or function (Table 3).

**Table 3:** Factors on Sequelae-free Result of Bone and Function

Variables	Sequelae-free Bone Result				Sequelae-free Function Result			
	Num of studies	Odds Ratio (95% CI)	*P* value	*I*^2^(%)	Num of studies	Odds Ratio (95% CI)	*P* value	*I*^2^(%)
Age	7	0.995(0.961 to 1.031)	0.789	0.0%	10	0.969(0.936 to 1.003)	0.075	0.0%
Previous Surgery Times	5	0.687(0.480 to 0.984)	0.040	37.5%	4	0.338(0.189 to 0.603)	0.000	0.0%
Bone defect	6	1.030(0.842 to 1.260)	0.776	0.0%	7	0.828(0.657 to 1.043)	0.109	3.2%
Length of Distraction	4	0.979(0.605 to 1.583)	0.930	49.6%	5	0.919(0.664 to 1.273)	0.613	14.8%
Time of External Fixation	4	0.977(0.924 to 1.032)	0.404	0.0%	4	0.899(0.825 to 0.979)	0.015	0.0%
Time of Bone Union	4	0.962(0.872 to 1.060)	0.431	0.0%	4	0.836(0.740 to 0.954)	0.004	0.0%

## Discussion

Infection relapse of tibial infectious nonunion and chronic osteomyelitis is common, because most causative bacteria are antibiotic-resistant, making it difficult to completely eradicate their populations with common prophylaxis [28, 29]. The affected patients tend to incur more than one surgery before achieving a successful infection-free result. Bone transportation techniques with external or hybrid fixator systems have been proved versatile to deal with this clinical challenge. However, the infection-free rates of these two fixator systems remain unknown and seldom compared. In the present meta-analysis, hybrid fixators were found to have a higher rate of infection recurrence compared to external fixators (Fig. 2).

In bone transportation technique, the choice of fixator systems depends on the treatment philosophy of long bone infection [17]. However, this philosophy has evolved in recent decades, from “the infection would burn on the fire of the bone regeneration,” by Ilizarov, to “the only cure for osteomyelitis is radical debridement,” by Cierny [30]. The later concept has greater amounts supportive evidence recently and is more widely accepted [31]. In most cases the infected tissue was extensively excised until live and bleeding bone (paprika sign), with which a large amount of bacterial burden would be removed. Based on this prerequisite it seems safe to implement internal fixators during or after the distraction period to overcome the disadvantage of external fixation [17]. However, the risk of infection recurrence comes along with internal fixators still exists. The reason is that debridement alone is not sufficient to sterilize the operative site. Small bacterial colonies can be displaced during the debridement procedure even under most careful cleaning [32]. If internal fixator is subsequently implanted, the residual bacteria on the device surface could form a biofilm, leading to infection recurrence. Consistently, in this research hybrid fixators showed potential inferior infection-free result than external fixators, indicating the internal fixators may not be appropriate in this specific scenario.

Similarly, Bose suggested that external fixators are safer than internal fixation for an infected nonunion fracture. Though all the infected bone and unhealthy enveloping soft tissue were completely excised, four of six patients had infection recurrence after internal fixation [21]. Liodakis found that in cases where an intramedullary rod was involved, there was a greater rate of infection recurrence than in cases of external fixation when dealing with infected post-traumatic tibia. He recommended the post-traumatic bone defects with chronic infection should only be fixed by external frame [8]. Besides providing a potential harbor for residual bacteria, the intramedullary nails also facilitate the spread of the pin tract infection along implants [33].To avoid internal-related infection recurrence, internal fixators can be applied at a later time, not immediately after debridement. In this way, when the distraction phase is over, the external fixators are removed and replaced by internal fixators. Zhang conducted early osteosynthesis with plate or intramedullary nail once two bone ends meet at the dock site. and no reinfection was noted [19]. However, Emara suggested this method still has risk of infection recurrence; in his study infection recurrence occurred in one patient who received early nailing, and the intramedullary nail was changed to an Ilizarov fixator until final union [18]. Last but not least, patients’ immunologic status has an impact on the infection-free rates. Within the hybrid fixator treatment group in Oh’s study, a diabetes mellitus patient was complicated with infectious recurrence [15]. Even though no statistical conclusion is draw due to the small sample size, patients with inferior immunologic function likely have a greater risk of infection recurrence and may be better suited for treatment by external fixator, which is safer.

Complete coverage of soft tissue is important to control and prevent infection. The early coverage of soft tissue may provide nutrition, obliterate dead space, facilitate local immunologic defense, and antibiotic delivery [22]. In most cases, soft tissue reconstruction was done empirically according to surgeons’ evaluation and preference. In the current review musculocutaneous flaps and skin grafts were the most commonly used methods to compensate for lost soft tissue and achieve satisfactory coverage (Table 1). However, due to the insufficient reported data, the hypothesis that soft reconstruction could increase infection-free rates, and the implementation of internal fixators could undermine the soft tissue, cause poor vascularity, and consequently reduce infection-free rates, remains to be investigated. Oh believed implantation of a locking plate would not compromise the surrounding soft tissue because of minimally invasive or percutaneous techniques [27]. Even though more thorough evidence is warranted to draw a conclusion for the role of soft tissue, proper reconstruction of surrounding soft tissue is still a great concern when surgical plans are made.

Despite these observations, the advantages of hybrid fixators are still remarkable. The external fixator could be removed once defect ends meet with combined fixation. This shortens the timeframe for potential distraction-related complications. Additionally, early removal of external fixators is more comfortable for the patients [17]. It is well known that the long-time external fixation imposes psychosocial hardships and disruption in daily lives to patients. The bulky external fixators interrupt activities of daily living (ADL) as well as leisure and sport activities [24]. Combining internal fixators facilitates the early return to ADL without the need for wearing external apparatus. Similarly, it can also allow early rehabilitation and prevent related joint stiffness [17]. Hybrid fixators demonstrated the highest success rate compared with external fixators alone for bone healing in the case of limb salvage of long bone defects [12]. However, for the infected cases, the higher potential risk of infection recurrence is more worrisome and serious than uncomfortableness. Notably, internal fixators used in all included studies were traditional internal implants without antibiotic surface modification. Antibiotic-coated implants have gained increasing interest recently. These novel surface coatings have been successfully used in the treatment of osteomyelitis and long bone infectious nonunion [28, 34–37]. This method could provide both infection control, or prophylaxis, and osseous stability simultaneously [28, 34]. Further research in this area is necessary towards a more comprehensive understanding of the long-term success of such surface coatings.

Infection recurrence leads to multiple operations [17, 38]. Many patients with tibia infection suffer from repeated surgeries before seeking the final bone transport treatment. In the present analysis, the number of previous operations is a predictive factor of prognosis for bony union (*p* = 0.04, Table 3) and functional restoration (*p* < 0.01, Table 3): a greater number of previous surgeries are correlated with a greater chance of sequelae. Several reason could explain this result. First, the repeatedly debridement and surgery would lead to prolonged hospital stays, loss of soft and bone tissue, impaired function of the affected limbs, increased pain, and a poor quality of life [39]. Additionally, the repeated operation could cause more scar tissue and subsequently poor soft tissue flexibility, which cause difficult exposure for the subsequent procedures [40, 41]. The consequent surgeries pose a high risk of poor outcome, as well as financial and psychological burdens to patients. Number of previous surgeries is a useful indicator to predict the treatment outcome for the patients with repeated revision. Therefore, it is important to meticulously design a surgery treatment plan and conduct limb salvage by an experienced multidisciplinary team in order to minimize the need for further unexpected operations.

The duration of external fixation has been suspected to be associated with a worse functional result. However, it is hard to make a definitive conclusion because of small sample sizes in previous research. In the present study, by pooling the IPD to increase sample size, the time of external fixation is found to be associated a greater risk of functional sequelae (*p* = 0.015, Table 3). In cases of extended duration of external fixation patients tend to suffer more joint stiffness, muscle dystrophy and significant pain. Additionally, more pin-related infections are also involved [6]. Hence, a shortened external frame time is necessary to reduce functional complications related to external fixators and improve patient outcomes [14, 16, 17]. To shorten external frame time of distraction, one option is to add more transport segments in large bone defects. Paley and Maar suggested trifocal bone transport when the bone defect is larger than 10 cm [24], while Rozbruch and Zhang set the criterion for trifocal transport at greater than 6 cm [42]. All trifocal patients in their studies had reduced distraction times. The trifocal bone transport could double the distraction speed because two-level osteotomies divided lengthening (and healing) into two locations [14]. To shorten the duration of dock consolidation, combining an internal implant could facilitate the early removal of external fixators and rehabilitation. However, there is a potential risk of infections recurrence, which should be considered in treatment and surgical planning. If the antibiotic-coated internal implants could achieve satisfactory control and prophylaxis of infection, it is an effective choice to manage cases of tibia infectious nonunion and osteomyelitis.

This is the first study to summary the infection-free results of bone transport techniques with external and hybrid fixators for tibia infectious nonunion and chronic osteomyelitis, and the first to determine factors which predict bone and function sequela. Here, hybrid technique involved traditional internal fixators showed more potential risk of infection recurrence than external groups. The number of previous operations and the duration of external fixation were confirmed associated with greater risk of sequelae. However, the results of this analysis should be interpreted carefully because of the limitations of this study. First, most of the included studies are retrospective with small sample sizes. Second, though the principle of the criterion of the ASIMI and Paley classifications are the same, in which classifications are graded by number of sequelae, difference exists in their criterion. Third, the included studies have obvious heterogeneity even though selection criteria were set. In the future prospective and large-scale clinical research is necessary to better understand the factors influencing patient outcomes.

## Conclusions

Bone transport technique is an established treatment to deal with segmental bone defects due to infection. Hybrid fixator system combining traditional internal and external fixators may be associated with a greater risk of infection recurrence; antibiotic-coated internal implants maybe a promising choice to circumvent this well-known issue. Additionally, we have demonstrated that number of previous operations as well as duration of external fixation are useful prognostic indicators for predicting outcomes. To achieve successful healing and functional results, meticulous surgical planning is necessary in order to avoid additional surgeries and long external fixation times.

## Abbreviations

AD: Aggregate Data
ADL: Activities of Daily Living
EF: External Fixator
IPD: Individual Patient Data
PRISMA-IPD: Preferred Reported Items for Systematic Reviews of Meta-Analyses Statement for Individual Patient Data
TSF: Taylor Spatial Frame
